# Efficacy of Racecadotril in a Patient Affected by a Therapy-Refractory VIPoma and Carcinoid Syndrome

**DOI:** 10.1210/jcemcr/luae177

**Published:** 2024-09-30

**Authors:** Jannes Boesenkoetter, Ina Ellrichmann, Björn Konukiewitz, Mark Ellrichmann, Dominik M Schulte

**Affiliations:** Department of Interdisciplinary Endoscopy, Medical Department 1, University Hospital Schleswig-Holstein, Campus Kiel, 24105 Kiel, Germany; Pediatric Gastroenterology, Department of Pediatrics, University Hospital Schleswig-Holstein, Campus Kiel, 24105 Kiel, Germany; Department of Pathology, University Hospital Schleswig-Holstein, Campus Kiel, 24105 Kiel, Germany; Department of Interdisciplinary Endoscopy, Medical Department 1, University Hospital Schleswig-Holstein, Campus Kiel, 24105 Kiel, Germany; Division of Endocrinology, Diabetes and Clinical Nutrition, Department of Internal Medicine I, University Hospital Schleswig-Holstein, Campus Kiel, 24105 Kiel, Germany; Institute of Diabetes and Clinical Metabolic Research, University Hospital Schleswig-Holstein, Campus Kiel, 24105 Kiel, Germany

**Keywords:** VIPoma, WDHA syndrome, carcinoid syndrome, racecadotril, neuroendocrine neoplasia

## Abstract

Neuroendocrine neoplasms (NENs) encompass a heterogeneous spectrum of tumors originating from the diffuse neuroendocrine cell system. Approximately 30% of NEN exhibit functional activity with clinical syndromes through hormone-mediated effects. Synchronous and metachronous functioning syndromes, resulting from the simultaneous release of distinct hormones, are exceptionally rare. Of note, hormonal excess syndromes can have a greater effect on patients’ morbidity and mortality than the tumor mass itself. We present the case of a 49-year-old male patient affected by an oligo-metastatic ileal NEN, concurrently demonstrating vasointestinal peptide (VIP) and serotonin excretion, complicated by pulmonary tuberculosis. After the first cycle of Lutetium-177-DOTATATE peptide-radio-receptor therapy, the patient developed a severe watery diarrhea, hypokalemia, and achlorhydria (WDHA) syndrome, despite receiving high-dose somatostatin analogues, everolimus, and telotristat ethyl, without any surgical options. The WDHA syndrome necessitated intensive-care-unit (ICU) admission with continual intravenous administration of electrolytes and fluids. With limited alternatives, an off-label intervention using the enkephalinase inhibitor racecadotril was initiated. After 5 days of treatment, the WDHA syndrome exhibited sufficient control, facilitating the patient's discharge from the ICU. This case report underscores racecadotril as an individualized, off-label treatment strategy for patients with severe VIPoma and serotonin-driven WDHA syndrome, where conventional therapeutic avenues have been exhausted.

## Introduction

Neuroendocrine neoplasms (NENs) comprise a heterogeneous group of tumors arising from diffuse endocrine cells causing intricate clinical syndromes through hormone secretion. Accounting for about 0.5% of newly diagnosed malignancies, neuroendocrine tumors are rare entities. About two-thirds of NENs affect the gastrointestinal tract, followed by manifestation in the respiratory tract [1].

Gastroenteropancreatic NENs (GEP-NENs) are characterized by a diversity of clinical symptoms contingent on their unique hormone secretion profiles and anatomic locations. Carcinoid syndromes, characterized by excessive serotonin production, result in watery diarrhea, flushing, hypotension, and bronchospasm. Conversely, pancreatic NENs present as insulinoma, gastrinoma, glucagonoma, or VIPoma, each displaying a spectrum of clinical symptoms. Apart from hereditary predispositions, encompassing multiple endocrine neoplasia syndromes or neurocutaneous syndromes, the concurrent occurrence of multiple hormone production types is exceedingly rare. In pancreatic NENs, metachronous functioning syndromes affect 3% to 6% of all patients [2, 3].

Treatment of metastatic GEP-NENs, without possible surgical therapeutic approaches, mainly focuses on targeting the somatostatin receptor. First-line therapeutic strategies comprise somatostatin analogues, followed by peptide radionuclide receptor therapy (PRRT) with lutetium (Lu)-177 DOTATATE, and everolimus drug therapy [4].

In the presented case, we describe the substantial symptomatic effect of the enkephalinase inhibitor racecadotril in a patient with metastatic GEP-NEN suffering from both carcinoid syndrome and VIPoma activity that proved refractory to standard therapeutic interventions.

## Case Presentation

In August 2022, a 49-year-old male patient, who recently migrated from Ukraine to Germany, had been referred to the European Neuroendocrine Tumor Society Centre of Excellence (ENETS CoE) at the University Hospital Schleswig-Holstein (UKSH), Campus Kiel, because of a progressive tumor burden of a known NEN and subsequent diarrhea with intermittent flush symptoms followed by significant weight loss.

**Figure 1.**
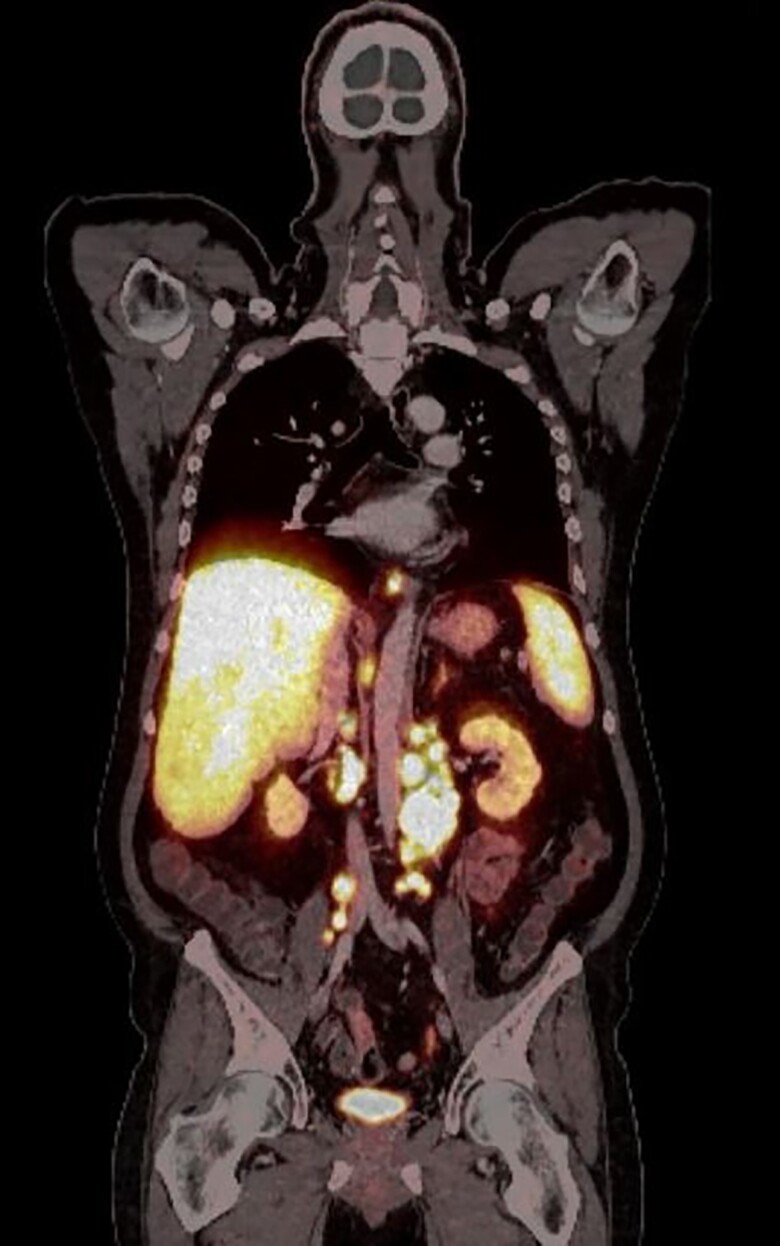
Positron emission tomography–computed tomography revealing lymphatic para-aortic and hepatic metastasis.

The NEN was initially diagnosed in 2017 in Ukraine with increased excretion of serotonin consistent with a carcinoid syndrome. Primary tumor had been suggested to be located in the ileum. A Ga68-DOTATATE-PET/CT revealed hepatic, lymphatic, and osseus metastasis at initial diagnosis. Lymphatic and hepatic metastasis are shown in Fig. 1.

The further patient history contained known hypertension as well as a partial adrenal insufficiency, iatrogenically caused by glucocorticoid therapy. Current drug therapy included the long-acting somatostatin analogue lanreotide 120 mg subcutaneously every 2 months combined with hydrocortisone 10 mg (2-1-0) orally.

## Diagnostic Assessment

At the first contact at our endocrinologic outpatient center, blood samples showed elevated neurone-specific enolase with 22.5 µg/L (normal reference range: < 12.5 µg/L), elevated chromogranin A with 622 ng/mL (normal reference range < 100 ng/mL), and elevated 5-hydroxyindoleacetic acid in 24-hour urine with 19.7 mg/24 hours (103.2 µmol/24 hours) (normal reference range, 2-9 mg/24 hours or 10.4-46.8 μmol/24 hours). Secretion greater than 15 mg or 50 μmol is considered compatible with the diagnosis of carcinoid syndrome. Because of the potential renal toxicity associated with PRRT, obligatory kidney scintigraphy had been performed prior to the first PRRT and showed a bilateral unimpaired renal function.

## Treatment

In response to significantly elevated laboratory results and symptomatic deterioration, the therapeutic strategy was adjusted accordingly. The medication regimen was intensified, incorporating lanreotide at a dosage of 120 mg administered subcutaneously every 2 weeks. Additionally, as a second-line intervention, everolimus was introduced at a daily dose of 5 mg.

**Figure 2.**
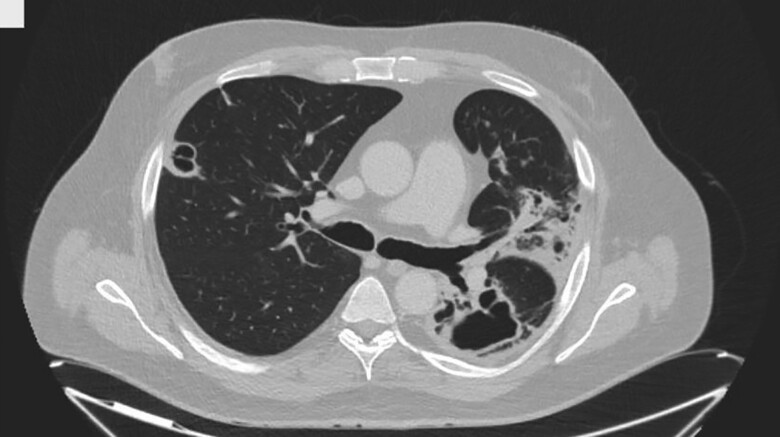
Chest computed tomography scan showing pulmonary tuberculosis with caverns connected to left main bronchus.

Furthermore, in January 2023, an initial PRRT was conducted, using 6.8 GBq Lu-177-DOTATATE. At the time of the first PRRT the patient still showed unchanged symptoms of watery diarrhea and progressive weight loss despite intensified drug therapy. Within 2 days post therapy, the patient manifested symptoms of fever reaching up to 39.5 °C and a progressing cough. A low-dose computed tomography (CT) scan of the lungs, shown in Fig. 2, revealed multiple caverns, highly indicative of disseminated lung tuberculosis. Sputum analysis confirmed an active Mycobacterium tuberculosis infection with multidrug-resistant tuberculosis (MDR-TB), demonstrating primary resistance to isoniazid and rifampicin. In accordance with German guidelines for MDR-TB treatment, the patient underwent specific tuberculosis treatment with bedaquiline 400 mg (1-0-0), pretomanid 200 mg (1-0-0), linezolid 600 mg (1-0-0), and moxifloxacin 600 mg (1-0-0).

Following the initial improvement in watery diarrhea and weight loss lasting for 5 weeks, symptoms once again worsened during TB treatment in March 2023. In April 2023, the second PRRT session was conducted after preceding kidney scintigraphy while maintaining ongoing TB treatment.

Due to the development of pancytopenia, everolimus was temporarily discontinued 2 weeks after the second PRRT session. By May 2023, symptoms progressively worsened, reaching up to 15 episodes of massive, watery diarrhea per day, accompanied by substantial hypokalemia and hyponatremia.

Owing to the uncontrollable diarrhea, the patient experienced acute renal failure and metabolic acidosis, necessitating intermittent hemodialysis and admission to our intensive care unit. Confronted with the therapy-refractory nature of the recognized carcinoid syndrome, comprehensive endocrinologic laboratory assessments were conducted, revealing markedly elevated fasting vasoactive intestinal peptide (VIP) serum levels exceeding 120 pmol/L (438 pg/mL) (normal reference range <20 pmol/L or <70 pg/mL). Subsequently, the patient was diagnosed with a NEN concurrently exhibiting features both of carcinoid syndrome and VIPoma functionality.

In retrospect the initial deterioration of watery diarrhea and weight loss in March 2023 was attributed to PRRT-related tumor decay with massive VIP secretion rather than to TB treatment.

During intensive care management, the administration of lanreotide was ceased and substituted with telotristat ethyl at a dosage of 250 mg (1-1-1), an inhibitor of tryptophan hydroxylases. Telotristat ethyl may be initiated as a potent second-line therapy in carcinoid syndrome when achieving adequate symptom control with somatostatin analogues is unattainable. Given the insufficient control of the aforementioned watery diarrhea after 3 days, drug therapy was further intensified by commencing continuous intravenous infusion of octreotide (300 µg per 24 hours). As the treatment progressed, the mode of octreotide administration was transitioned to subcutaneous application with octreotide 100 µg (1-1-1). During combined drug therapy and supportive intensive care treatment, the massive watery diarrhea and consecutive hypokalemia and hyponatremia stabilized for 3 weeks. Renal function improved and intermittent hemodialysis had been paused, but renal function stayed too compromised to continue PRRT.

In June 2023, a recurrence of severe, watery diarrhea ensued, accompanied by considerable hypokalemia, hyponatremia, and renal insufficiency requiring dialysis. Due to the absence of additional intensification options for the already highly dosed second-line drug therapy, an off-label treatment endeavor was initiated with the potent enkephalinase inhibitor racecadotril at a dosage of 100 mg (1-1-1). Within 5 days, a marked reduction in the frequency of watery diarrhea was observed and resulted in clinical stabilization, allowing for the ongoing management both of the TB and NEN therapy in our TB ward. By August 2023, renal function, as well as potassium and sodium levels, normalized under rigorous monitoring of laboratory values.

Because of the severely impaired general state and the difficult differentiation between diffuse lymphatic para-aortic and hepatic metastasis or TB-affected lymph nodes, no surgical debulking procedures were performed in this patient.

## Outcome and Follow-up

With sustained administration of racecadotril, octreotide was successfully discontinued in September 2023, and the recommencement of PRRT became feasible due to improved renal function and an enhanced overall health condition. By the end of September, discharge from the TB ward was finally attainable following 3 consecutive negative sputum analyses. During this period, oral drug therapy comprising telotristat ethyl 250 mg (1-1-1) and racecadotril 100 mg (1-1-1) consistently maintained stable control over the symptoms of carcinoid syndrome and VIPoma.

Consecutively, the next PRRT treatment was scheduled for December 2023. As of July 2024, no further adverse events occurred under the aforementioned drug treatment, especially under continued long-term treatment with racecadotril.

## Discussion

Racecadotril, the prodrug of its active metabolite thiorphan, exerts its effects by inhibiting the enzyme neutral endopeptidase. Among its primary substrates are enkephalins and neuropeptide Y, with enkephalins being particularly notable for their potent antisecretory effects without affecting gut motility. Research has demonstrated that racecadotril diminishes pathological hypersecretion without affecting basal gut secretion. Numerous experimental and clinical studies have revealed a significant reduction in stool frequency and weight in acute infectious diarrhea both in adults and children following the administration of racecadotril. Additionally, racecadotril has proven effective in chemotherapy-associated diarrhea, and owing to its negligible effect on gut motility, adverse events such as abdominal pain, constipation, nausea, and vomiting are comparable to those observed with a placebo. Moreover, racecadotril demonstrates efficacy in the symptomatic therapy of AIDS-associated therapy-refractory diarrhea [5‐7].

To our knowledge this is the first case report showing the potent effect of racecadotril in therapy-refractory diarrhea caused by a NEN.

Analyzing the pharmacodynamics of racecadotril in relation to the pathophysiology of carcinoid syndrome and VIPoma may provide insights into the observed symptomatic effects in this particular case.

In carcinoid syndrome, excessive serotonin excretion induces hyperperistalsis, resulting in diminished reabsorption of water and electrolytes. The elevated concentrations of electrolytes and increased luminal water content contribute to the development of diarrhea in affected patients [8, 9].

In the context of VIPoma, a different pathophysiological mechanism is encountered. The characteristic watery diarrhea, hypokalemia, and achlorhydria syndrome (WDHA), also referred to as pancreatic cholera, arises from augmented VIP excretion by tumor cells. The consequential severe diarrhea predominantly results from VIP-mediated stimulation of VPAC1 receptors situated in the intestinal mucosa.

Hyperstimulation of VPAC1 receptors induces an extensive secretion of chloride anions and water into the intestinal lumen, leading to the manifestation of severe watery diarrhea. The pathomechanism of this G protein–coupled pathway, involving adenylyl cyclase and protein kinase A activation, bears similarities to the effect of the cholera toxin [9, 10].

Even though experimental studies showed a significant decrease in water and electrolyte hypersecretion caused by the cholera toxin after racecadotril application, a double-blind, randomized, controlled clinical trial failed to establish any advantages of racecadotril over placebo in cholera patients [11, 12]. Consequently, the precise mechanistic effect of racecadotril in the presented patient case remains uncertain. Focusing on the pharmacodynamics of racecadotril on the hypersecretory component of diarrhea, we assume a VIP-dependent clinical effect rather than an improvement in possible serotonin-associated diarrhea.

Complicated by the MDR-TB, the subsequent postponement of further PRRT sessions must be regarded as a pivotal factor contributing to therapy resistance in NEN treatment. Due to the rapidly evolving clinical condition of the patient in question, adherence to predefined therapy intervals proved unattainable, resulting in the diminished effectiveness of PRRT.

Regarding the safety of a long-term usage of racecadotril in chronic diarrhea, studies are still missing. Racecadotril has an approval for short-term use in acute diarrhea only. Facing the off-label treatment in the described patient, no adverse events, a stable disease, and stable electrolytes are observed after 13 months of treatment.

In summary, we attribute the episodes of severe diarrhea mainly to the hormone excretion of the 2 NEN entities. Particularly in patients with VIPoma, we suggest a therapy approach with racecadotril as a potential off-label use to control massive watery diarrhea. Numerous studies have reported diverse effects of racecadotril, but agreed about the low potential for adverse events. As demonstrated, racecadotril could provide a benefit in symptom control of selected patients.

## Learning Points

- Neuroendocrine tumors with synchronous and metachronous functioning syndromes caused by simultaneous release of different hormones are extremely rare.
- In a patient with a neuroendocrine neoplasm affected by severe symptoms despite an intensified guideline-based therapy, a second NEN entity/functionality should be considered.
- Racecadotril provides a benefit in symptomatic treatment of severe diarrhea, especially in VIPoma patients.

## Data Availability

Original data generated and analyzed during this study are included in this published article.

## Abbreviations

GEP-NEN: gastroenteropancreatic neuroendocrine neoplasm
Lu: lutetium
MDR-TB: multidrug-resistant tuberculosis
NEN: neuroendocrine neoplasm
PRRT: peptide radionuclide receptor therapy
VIP: vasointestinal peptide
WDHA: watery diarrhea, hypokalemia, and achlorhydria
